# Evaluation of sarcopenia in female patients with fibromyalgia syndrome: an ultrasonographic approach

**DOI:** 10.3389/fmed.2025.1680355

**Published:** 2025-09-09

**Authors:** Salih Karvanli, Serdar Karakose, Hatice Ugurlu

**Affiliations:** ^1^Department of Physical Medicine and Rehabilitation, Kirikhan State Hospital, Hatay, Türkiye; ^2^Department of Radiology, Faculty of Medicine, Necmettin Erbakan University, Konya, Türkiye; ^3^Department of Physical Medicine and Rehabilitation, Faculty of Medicine, Necmettin Erbakan University, Konya, Türkiye

**Keywords:** fibromyalgia, muscle, sarcopenia, ultrasonography, dynapenia

## Abstract

**Objectives:**

The aim of this study is to evaluate sarcopenia in female patients with Fibromyalgia Syndrome (FMS), according to diagnostic algorithms, in terms of total and regional muscle mass measurements.

**Methods:**

The study included 60 female patients diagnosed with FMS (mean [M] = 47.78 years, Standard deviation [SD] = 4.72 years) and 30 healthy women (M = 49.8 years, SD = 4.8 years). The ages and anthropometric measurements were noted, and participants evaluated using the Widespread Pain Index, Symptom Severity Scale, Visual Analog Scale, Fatigue Severity Scale, Beck Depression Inventory, International Physical Activity Questionnaire, and the Fibromyalgia Impact Questionnaire.

**Results:**

The hand grip strength and gait speed values of the patient group were found to be lower than those of the control group, and the chair stand test value higher than that of the control group. There was a statistically significant difference between the groups (*p* < 0.01). Two individuals in the patient group were classified as having sarcopenia according to the ISarcoPRM algorithm. No statistically significant difference was found between the patient group and the control group in terms of anterior thigh muscle thickness, sonographic thigh adjustment ratio, and skeletal muscle mass index (*p* = 0.897, *p* = 0.829, *p* = 0.706).

**Conclusion:**

The female patients with FMS were dynapenic, which means a loss of muscle function without a loss of muscle mass.

## 1 Introduction

Fibromyalgia Syndrome (FMS) is a central sensitization syndrome characterized by chronic widespread musculoskeletal pain that is accompanied by fatigue, sleep disturbance, cognitive, and somatic symptoms (1). The presence of small fiber polyneuropathy has been reported in patients with FMS (2), and recent studies have suggested that inflammation may also play a role in the etiology (3, 4).

Sarcopenia is defined as a loss of muscle strength and mass. Although commonly associated with aging, sarcopenia can also be caused by various factors including biological changes in muscle structure, neurological issues, hormonal imbalances, immunological factors, poor nutrition, and physical inactivity. Recent studies extend beyond traditional studies of age-related decline in muscle mass and function (5) to highlight that sarcopenia may also affect younger individuals.

Several studies have explored the presence of sarcopenia in patients with FMS (6, 7). Recent systematic review has used the European Working Group on Sarcopenia in Older People (EWGSOP) diagnostic algorithm for exploring the presence of sarcopenia based on total mass measurement, however presence of sarcopenia could not be demonstrated (8).

There are currently limited options for managing sarcopenia with prevention and early diagnosis remaining the most effective strategies. There have been recent explorations into the potential role of ultrasonography in diagnosing sarcopenia, particularly in comparison with other diagnostic techniques (9). Recent studies on the Sonographic Thigh Adjustment Ratio (STAR) revealed that the most pronounced and significant decrease in muscle thickness with age occurs in the anterior thigh muscle, specifically within the quadriceps femoris (10). In this respect, it may be meaningful to evaluate sarcopenia by regional muscle measurement with ultrasonography, especially in middle-aged patients.

The International Society of Physical and Rehabilitation Medicine Special Interest Group on Sarcopenia (ISarcoPRM) algorithm offers a functional perspective on sarcopenia for diagnosis using the STAR formula. It is based on regional measurements of the most commonly and initially affected muscles by ultrasonography. This method could help clinicians identify sarcopenia earlier (11).

We aimed to investigate sarcopenia status in female patients diagnosed with FMS by using both total and regional muscle mass measurements. A secondary aim was to investigate the relationship between disease-related factors and sarcopenia parameters in FMS patients.

This article was produced from the thesis: Fibromiyalji Sendromlu Kadın Hastalarda Sarkopeni Değerlendirmesi (Completed).

## 2 Patients and methods

The power analysis was performed with G*Power version 3.1.9.7 (Heinrich-Heine-Universität Düsseldorf, Düsseldorf, Germany). The sample size calculation was based on an independent samples t-test (two-tailed). Considering an alpha of 0.05, a size effect of 0.738, and study power of 80%, the minimum number of participants needed was calculated as 68 (23 in the control group and 45 in the patient group). Considering that there may be missing data, 90 participants (30 in the control group and 60 in the patient group) were included in the study. The sample size was calculated according to the hand grip strength test results of the patient and control groups of the reference study (7).

### 2.1 Participant selection

The recruitment of participants was done from the outpatient clinic from the Department of Physical Medicine and Rehabilitation at Necmettin Erbakan University Meram Faculty of Medicine Hospital. The healthy control group consisted of subjects among health care workers and other medical staff from Necmettin Erbakan University Meram Faculty of Medicine Hospital on a voluntary basis.

#### 2.1.1 Inclusion criteria

**Figure 1:**
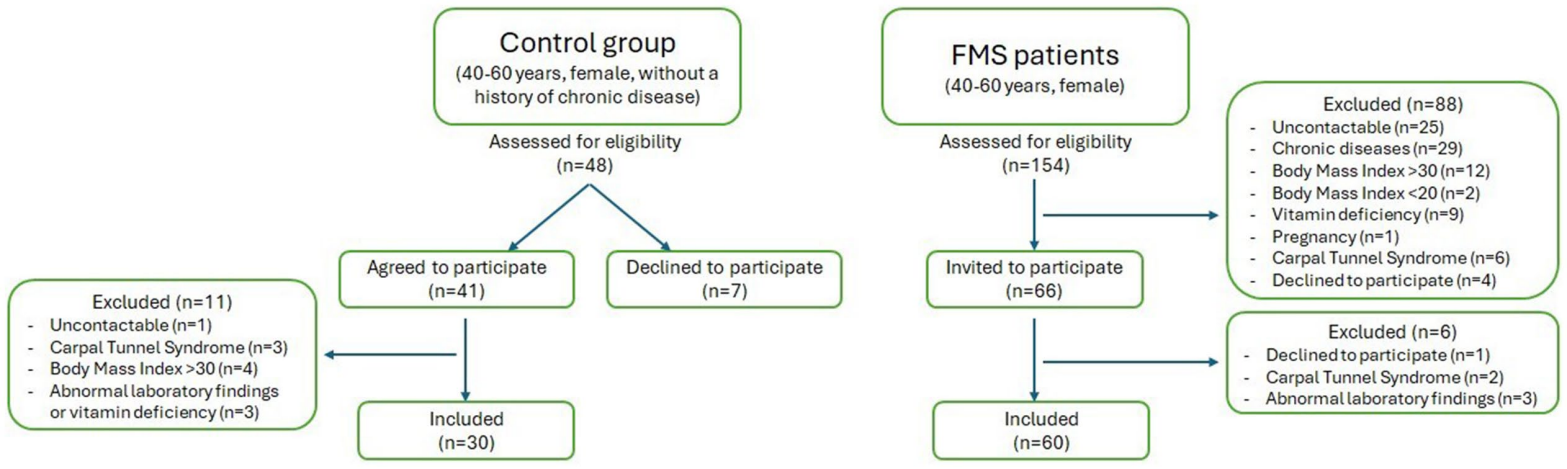
The flow diagram of participants.

The study comprised 60 female patients aged 40–60 years who were diagnosed with FMS, according to the 2016 American College of Rheumatology (ACR) criteria (12). Patients in FMS group did not have other chronic diseases or other rheumatologic diseases. The study was conducted between February 1 and December 31st, 2023. An additional 30 healthy female volunteers in the same age range were included for comparison (Figure 1).

#### 2.1.2 Exclusion criteria

Exclusion criteria included individuals being outside the specified age range; having a history of disease of more than 2 weeks in the past 6 months including neurological deficits, respiratory diseases, cardiovascular diseases, renal or liver failure, osteoporosis, moderate to severe carpal tunnel syndrome, malignancies, other rheumatic diseases, active synovitis, bursitis, inflammation, immobilization; steroid use exceeding 40 mg/day for more than 1 week in the past 3 months; pregnant or breastfeeding women; obese patients (Body Mass Index [BMI] > 30); underweight patients (BMI < 20); patients with a severe vitamin D or B12 deficiency; and individuals with a history of hospitalization for mood disorders.

### 2.2 Evaluation of the participants

All assessment methods were administered to participants in both groups in the same order. The dominant side was determined by asking participants which side they preferred for their daily activities (writing, throwing, kicking, etc.).

The procedures for each of the tests within the assessment methods were demonstrated to the participants. Before the formal tests, each participant was allowed one practice trial. All tests were repeated three times for each participant, and the mean of the results was recorded.

A digital stopwatch was used to record the time in seconds for the Chair Stand Test (CST), Gait Speed and Timed Up and Go (TUG).

#### 2.2.1 Evaluation form

Routine laboratory tests, including measurements of vitamin D (ng/mL) and vitamin B12 (ng/L), were conducted. Participants were excluded based on the eligibility criteria. The duration of FMS was recorded.

Anthropometric measurements were taken, including height (m), weight (kg), waist circumference (cm), hip circumference (cm), upper middle arm circumference (cm), and calf circumference (cm) on the dominant side. Body Mass Index (BMI) was calculated using the formula kg/m^2^. Standardized procedures were followed for all anthropometric measurements.

The following evaluations were used to evaluate the participants: Widespread Pain Index (WPI), Symptom Severity Score (SSS), Visual Analog Scale (VAS), Fatigue Severity Scale (FSS), Beck Depression Inventory (BDI), International Physical Activity Questionnaire (IPAQ), and Fibromyalgia Impact Questionnaire (FIQ).

The FSS assesses the severity of fatigue and its effect on a participant’s activities and lifestyle (13).

The BDI is a scale measures characteristic attitudes and symptoms of depression (14).

The physical activity level of the participants was evaluated using the International Physical Activity Questionnaire (IPAQ) (15). The IPAQ scores were categorized as follows: IPAQ-1, IPAQ-2, and IPAQ-3 scores were totaled to calculate the total IPAQ score, while the IPAQ-4 value, which reflects sedentary behavior, was calculated separately.

The FIQ assesses the impact of FMS on a participant’s physical function, job difficulty, depression, anxiety, sleep, pain, stiffness, fatigue, and well-being. It is a self-administered 10-item questionnaire with scores ranging from 0 to 100, where higher scores indicate a greater impact of FMS on the participant (16).

#### 2.2.2 Evaluation methods

##### 2.2.2.1 Hand grip strength

Hand grip strength (kg) was measured using the SAEHAN SH5001 Hydraulic Hand Dynamometer. Participants were instructed to squeeze the dynamometer as hard as they could using their dominant hand while seated. The elbow was flexed at 90 degrees, the forearm was in a neutral position, and the wrist was dorsiflexed between 0 and 30 degrees (17).

##### 2.2.2.2 Chair stand test

Participants were instructed to sit upright on a chair of standard height with their arms crossed in front of their chest. They were then asked to stand up and sit back down as quickly as possible without using their hands and then repeat this cycle. The test was stopped after the fifth repetition and the time taken to complete the test was recorded in seconds (17).

##### 2.2.2.3 Bioelectrical impedance analysis

Total muscle mass was estimated using the TANITA BC-418 MA device. The muscle mass values for the four extremities and the trunk were totaled to obtain the Total Skeletal Muscle Mass (SMM) (17). The Skeletal Muscle Index (SMI) was calculated using the formula: SMI = SMM / height^2^ (kg/m^2^).

##### 2.2.2.4 Ultrasonography

For regional muscle mass assessment, the anterior thigh muscle thickness on the dominant side was measured using ultrasonography. The ultrasonography device and a 4–15 MHz linear probe were utilized for this measurement (Esaote MyLab™X7, Genova, Italy).

**Figure 2:**
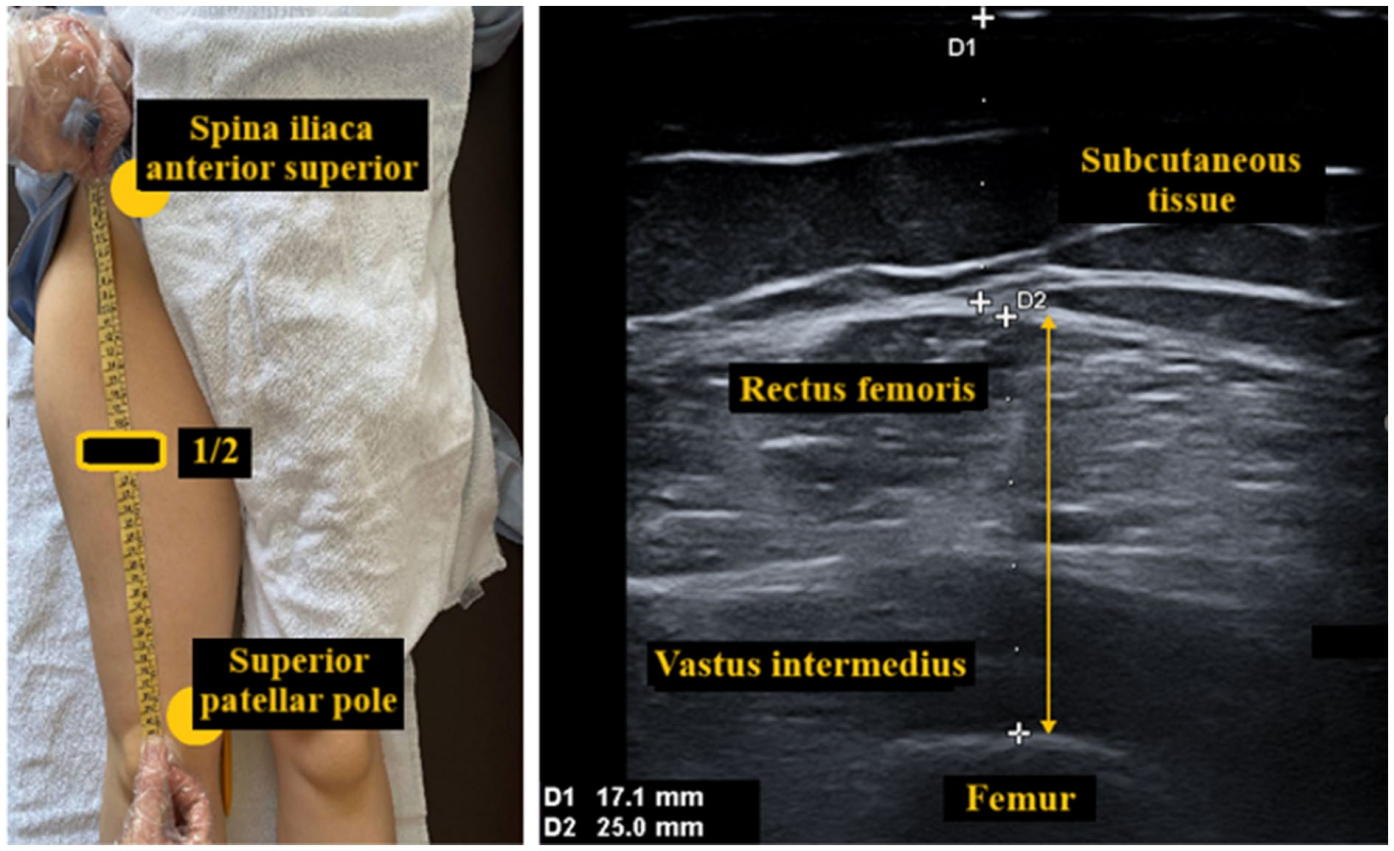
Evaluation of anterior thigh muscle thickness by ultrasonography.

Participants were evaluated in a supine position with arms and legs extended and muscles completely relaxed. The exact midpoint between the spina iliaca anterior superior and the upper pole of the patella was determined as the measurement site. An ultrasound probe was placed axially at 90 degrees perpendicular to the muscle fibers. Images were obtained without compression with the use of a generous amount of gel. Anterior thigh muscle thickness (the sum of rectus femoris and vastus intermedius thicknesses) was defined as the distance between the outer fascia of the rectus femoris muscle and the femoral periosteum. The placement of the probe on the anterior thigh and measurement of muscle thickness are shown in Figure 2.

Ultrasound measurements were performed by an experienced radiologist who was blinded to the participants’ group assignments. The measurement was repeated three times for each participant, and the mean of the measurements was noted. STAR was calculated by dividing the obtained anterior thigh muscle thickness by the participant’s BMI (10, 11).

##### 2.2.2.5 Gait speed

Participants were asked to walk at their own pace on a flat 4-meter surface (17).

##### 2.2.2.6 Timed up and go test

Participants were instructed to stand up from their chair, walk a distance of 3 meters in a straight line, return to the chair, and then sit down again. The time taken to complete this task was recorded (17).

#### 2.2.3 Reference values used in the evaluation

After all tests and measurements had been completed, both groups were evaluated for sarcopenia using the EWGSOP2 (17) and ISarcoPRM (11) algorithms.

The first step of the EWGSOP2 algorithm involves the SARC-F questionnaire. It is currently recommended for screening of sarcopenia in older adults. However, this step was omitted due to the age range of the participants in our study. For the parameters in the algorithm, regional thresholds determined according to the normal population are recommended whenever possible (17). Accordingly, a hand grip strength threshold of <22 kg was used, based on a Turkish population reference study, instead of the <16 kg threshold typically used for women (18). In the Chair Stand Test, a time >15 s was accepted as the threshold value specified in the algorithm for women. However, while the EWGSOP2 algorithm did not provide threshold values for Skeletal Muscle Mass (SMM) and Skeletal Muscle Index (SMI), thresholds were established for Appendicular Skeletal Muscle (ASM) and Appendicular Skeletal Muscle Index (ASMI). In the Turkish population reference study, a threshold value for SMI was recommended. Accordingly, in our study, 7.4 kg/m^2^ was used as the threshold value for SMI in women when evaluating muscle mass with BIA (18). According to the algorithm, a gait speed of ≤0.8 m/s and a time of ≥20 s for the TUG were accepted as the threshold values for women.

With the ISarcoPRM algorithm, participants were evaluated for sarcopenia based on regional muscle mass measurements. For women, the following threshold values were used (11): hand grip strength <19 kg, chair stand test ≥12 s, STAR <1.0, and gait speed ≤0.8 m/s.

Participants with loss of muscle strength and muscle mass were considered to have sarcopenia, and participants with loss of muscle strength but no evidence of loss of muscle mass were considered to have dynapenia.

### 2.3 Statistical analysis

The data obtained in our study was analyzed using SPSS 25 (Statistical Package for the Social Sciences, version 25). Normal distribution was assessed using the Kolmogorov–Smirnov test. Descriptive statistics are presented as median, 25th percentile, 75th percentile, mean, and standard deviation for continuous variables, and as frequency and percentage for categorical variables. In order to compare two independent groups with continuous variables, the independent samples t-test was used for normally distributed data, while the Mann–Whitney U test was used for non-normally distributed data. Categorical data was analyzed using Pearson’s Chi-Square and Fisher’s Exact tests. The relationships between continuous variables were examined using Pearson and Spearman correlation analyses. Correlation coefficients were interpreted as follows (19): 0–0.20 (weak correlation), 0.21–0.40 (low/moderate correlation), 0.41–0.60 (moderate correlation), 0.61–0.80 (significant/strong correlation), and 0.81–1.0 (near perfect correlation). A *p*-value of <0.05 was considered statistically significant.

## 3 Results

There was no statistically significant difference between the control and patient groups regarding age, height, weight, BMI, waist circumference, hip circumference, upper mid-arm circumference, and calf circumference (*p* > 0.05; Table 1).

**Table 1:** Comparison of age and the anthropometric data of the groups.

Variables	Group		
	Control	Patient	*p*
Age (years)	49.8 ± 4.8	47.78 ± 4.72	^a^0.068
Height (m)	1.59 ± 0.06	1.61 ± 0.06	^a^0.142
Weight (kg)	65.71 ± 6.47	67.96 ± 8.49	^a^0.205
BMI (kg/m^2^)	26.12 ± 2.48	26.3 ± 2.58	^a^0.748
Waist circumference (cm)	86.8 ± 8.23	88.39 ± 8.8	^a^0.411
Hip circumference (cm)	101.8 ± 5.9	103.7 ± 7.51	^a^0.230
Upper middle arm circumference (cm)	29.5 (27–30.5)	30 (28–32)	^b^0.065
Calf circumference (cm)	34.98 ± 1.77	35.96 ± 3.18	^a^0.122

Mean ± SD, median (25th–75th percentile).

a Independent sample *t* test.

b Mann Whitney U test.

There was no statistically significant difference between the groups in terms of IPAQ total scores, indicating that both groups were similar in terms of physical activity levels. However, IPAQ-4 scores were higher in the patient group with a statistically significant difference observed between the groups, suggesting that a sedentary lifestyle was more common with the patient group (Table 2).

**Table 2:** Comparison of scale scores and the vitamin levels of the groups.

Variables	Group		
	Control	Patient	*p*
Follow-up period (month)	-	36 (12–72)	**-**
WPI	-	8 (7–10)	**-**
SSS	-	9 (8–10)	**-**
VAS	-	8 (6–8)	**-**
FSS	-	5.11 ± 1.25	**-**
BDI	-	16 (11–20)	**-**
IPAQ Total	473.3 (278–946)	633 (266.3–1.144)	^b^0.758
IPAQ-4	1,260 (840–2.100)	2,100 (1680–2.950)	^**b**^0.001^*^
FIQ	-	55.29 ± 14.51	**-**
Vitamin D (ng/ml)	18.42 (15.1–24.8)	22.2 (17.75–25.9)	^b^0.088
Vitamin B12 (ng/L)	362 (296–483)	380 (290–522)	^b^0.659

BDI, Beck Depression Inventory; FIQ, Fibromyalgia Impact Questionnaire; FSS, Fatigue Severity Scale; IPAQ, International Physical Activity Questionnaire; SSS, Symptom Severity Score; VAS, Visual Analog Scale; WPI, Widespread Pain Index. Mean ± SD, median (25th–75th percentile).

*p < 0.01. Values in bold indicate statistical significance.

b Mann Whitney U test.

In the study, the hand grip strength and gait speed values of the patient group were lower than those of the control group, while the CST value was higher than that of the control group. There was a statistically significant difference between the groups (*p* < 0.01; Table 3).

**Table 3:** Comparison of the sarcopenia parameters of the groups.

Variables	Group		
	Control	Patient	*p*
Hand grip (kg)	24 (23–26.5)	21 (20–25)	^**b**^0.003^*^
CST (sec)	9.12 ± 0.89	11.22 ± 2.03	^**a**^<0.001^*^
Anterior thigh muscle thickness (mm)	33.18 ± 4.13	33.32 ± 5.37	^**a**^0.897
STAR	1.28 ± 0.19	1.27 ± 0.19	^a^0.829
SMI (kg/m^2^)	9.89 ± 0.58	9.84 ± 0.63	^a^0.706
Gait speed (m/s)	1.12 (1.01–1.26)	1.01 (0.94–1.06)	^**b**^0.001^*^
TUG (sec)	8.52 ± 0.86	8.7 ± 1.26	^a^0.466

CST, Chair Stand Test; SMI, Skeletal Muscle Index; STAR, Sonographic Thigh Adjustment Ratio; TUG, Timed Up and Go Test. Mean ± SD, median (25th–75th percentile); *p < 0.01. Values in bold indicate statistical significance.

a Independent sample *t* test.

b Mann Whitney U test.

No statistically significant difference was found between the patient group and the control group in terms of anterior thigh muscle thickness, STAR, and skeletal muscle mass index (*p* = 0.897, *p* = 0.829, *p* = 0.706; Table 3).

There was a negative low/moderate correlation between follow-up time and SMI (Table 4).

**Table 4:** Evaluation of the relationship between the follow-up period, scale scores, vitamin levels and sarcopenia parameters in the FMS group.

Variables		Hand grip	CST	Anterior thigh muscle thickness	STAR	SMI	Gait speed	TUG
Follow-up period (months)	R	−0.053	−0.116	−0.147	0.013	−0.283	0.074	0.245
	P	0.686	0.377	0.261	0.923	**0.029**	0.576	0.059
WPI	R	−0.065	0.288	−0.027	−0.263	0.023	−0.355	0.212
	P	0.624	**0.026**	0.836	**0.042**	0.859	**0.005**	0.104
SSS	R	0.003	0.332	−0.053	−0.103	0.042	−0.132	0.104
	P	0.984	**0.009**	0.690	0.435	0.749	0.315	0.427
VAS	R	−0.151	−0.146	−0.002	−0.010	−0.074	−0.076	0.078
	P	0.249	0.264	0.990	0.938	0.576	0.562	0.554
FSS	R	−0.111	0.285	−0.070	0.023	−0.185	−0.084	0.138
	P	0.397	**0.027**	0.595	0.859	0.156	0.522	0.292
BDI	R	0.016	0.438	0.098	0.042	0.141	−0.189	0.150
	P	0.906	**0.000**	0.457	0.752	0.283	0.148	0.254
FIQ	R	−0.189	0.345	−0.033	−0.024	0.041	−0.145	0.142
	P	0.148	**0.007**	0.802	0.858	0.756	0.271	0.278
Vitamin D	R	−0.079	−0.318	0.054	0.105	−0.082	0.015	−0.012
	P	0.551	**0.013**	0.679	0.426	0.532	0.907	0.929
Vitamin B12	R	−0.078	−0.134	−0.166	−0.033	−0.134	−0.101	−0.018
	P	0.552	0.306	0.205	0.800	0.308	0.441	0.889

BDI, Beck Depression Inventory; CST, Chair Stand Test; FIQ, Fibromyalgia Impact Questionnaire; FSS, Fatigue Severity Scale; SMI, Skeletal Muscle Mass Index; SSS, Symptom Severity Score; STAR, Sonographic Thigh Adjustment Ratio; TUG, Timed Up and Go Test; VAS, Visual Analog Scale; WPI, Widespread Pain Index. Pearson and Spearman correlation analysis. Values in bold indicate statistical significance.

A positive low/moderate correlation was observed between SMI and CST; a negative low/moderate correlation between SMI and STAR and gait speed; a positive low/moderate correlation between SSS and CST; a positive low/moderate correlation between FSS and CST; a positive moderate correlation between BDI and CST; and a positive low/moderate correlation between FIQ and CST (Table 4).

A negative low/moderate correlation was found between vitamin D level and CST value (Table 4).

In the study, 13.33% (*n* = 4) of the control group and 53.33% (*n* = 32) of the patient group were identified as having probable sarcopenia according to the EWGSOP2 diagnostic algorithm. While none of the participants in the control group were identified as having probable sarcopenia according to the ISarcoPRM diagnostic algorithm, 35% (*n* = 21) of the patient group were identified as having probable sarcopenia. The frequency of probable sarcopenia, according to both the EWGSOP2 and ISarcoPRM algorithms, was statistically significantly higher in the patient group than in the control group (*p* < 0.01; Table 5).

**Table 5:** The sarcopenia status of participants according to EWGSOP2 and ISarcoPRM algorithms.

Variables		Group		
		Control	Patient	*p*
Possible sarcopenia (EWGSOP2)	No	26 (86.67)	28 (46.67)	^**a**^<0.001*
	Yes	4 (13.33)	32 (53.33)	
Sarcopenia (EWGSOP2)	No	30 (100)	60 (100)	
	Yes	0 (0)	0 (0)	
Possible sarcopenia (ISarcoPRM)	No	30 (100)	39 (65)	^**a**^<0.001*
	Yes	0 (0)	21 (35)	
Dynapenia/Sarcopenia (ISarcoPRM)	None	30 (100)	39 (65)	^**b**^0.001*
	Dynapenia	0 (0)	19 (31.67)	
	Sarcopenia	0 (0)	2 (3.33)	
Severe sarcopenia	No		2 (100)	
	Yes		0 (0)	

EWGSOP, European Working Group on Sarcopenia in Older People; ISarcoPRM, International Society of Physical and Rehabilitation Medicine Special Interest Group on Sarcopenia.*p < 0.01. Values in bold indicate statistical significance.

a Pearson chi-square test.

b Fisher’s Exact test.

No individuals were identified as having sarcopenia according to the EWGSOP2 diagnostic algorithm (Table 5).

In the patient group, 90.48% (*n* = 19) of those identified as having probable sarcopenia according to the ISarcoPRM diagnostic algorithm were classified as having dynapenia, while two individuals were classified as having sarcopenia. The frequency of dynapenia in the patient group according to the ISarcoPRM algorithm was statistically significantly higher than in the control group (*p* < 0.01; Table 5).

## 4 Discussion

In our study, there were no participants with sarcopenia according to the EWGSOP2 algorithm, while sarcopenia was detected in two individuals in the patient group according to the ISarcoPRM algorithm. This supports the view that the ISarcoPRM algorithm, which is based on regional muscle mass measurement with ultrasonographic evaluation, provides a more strategic view of sarcopenia compared to the EWGSOP2 algorithm, which is based on total muscle mass measurement. For the diagnosis of sarcopenia, demonstrating a loss of muscle mass is essential. However, there was no statistically significant difference between the patient group and the control group in terms of anterior thigh muscle thickness and STAR. In this sense, we propose the presence of dynapenia in the light of results of our study. This situation is more likely to arise from pathologies other than sarcopenia in relatively young patients. While dynapenia is defined as loss of muscle strength, sarcopenia is defined as loss of muscle strength and mass (20).

In another study, 50 fibromyalgia patients and 50 healthy individuals between the ages of 25–65 years were evaluated with the ISarcoPRM algorithm, and sarcopenia was detected in 20 participants in the patient group and six participants in the control group (21). Compared to our study, the detection of sarcopenia in more patients and the detection of sarcopenia even in the healthy group may be due to the subjectivity of the ultrasonographic evaluation. As a matter of fact, it is possible to obtain larger or smaller values in muscle thickness depending on the compression that can be applied with the probe. This may have caused the number of participants with sarcopenia to be higher in the mentioned study. In our study, ultrasonographic evaluation was performed by an experienced radiologist who had no way of knowing which group the participants were in, without applying any compression other than the weight of the probe.

In studies comparing hand grip strength between patients diagnosed with FMS and healthy groups, it has been consistently shown that hand grip strength is lower in the patient group (6, 7, 22, 23). Our study yielded results that are consistent with findings in the literature. Additionally, it is important to note that lower extremity muscle strength tends to decline earlier and more significantly than upper extremity muscle strength (24). Therefore, relying solely on hand grip strength can lead to misclassification as it may not adequately reflect the loss of lower extremity muscle strength (25). It is for this reason that it is crucial to incorporate CST, which serves as an indicator of lower extremity muscle strength, particularly in the quadriceps muscle group. This test is also a performance test and provides valuable information about muscle function. In previous studies, participants evaluated with the CST demonstrated lower performance in the patient group compared to the healthy group (23, 26). Our study found similar results, with the patient group showing lower performance based on CST scores. Decreased muscle function observed in patients with FMS may be attributed to factors such as pain, motivation level, and changes in muscle structure.

The BIA method was selected for use in this study due to its affordability, ease of use in practice, and overall convenience. Previous studies comparing patients diagnosed with FMS to control groups have found no significant difference in SMI values obtained using BIA (6, 7). Consistent with these findings, this study also did not reveal a statistically significant difference.

There are studies where patients with FMS were evaluated using regional muscle measurements via ultrasonography (USG). In one study, it was noted that all muscle thicknesses evaluated were significantly lower in the patient group (23). In another study that assessed the thickness of various upper and lower extremity muscles, it was reported that both upper and lower extremity muscles were affected in the patient group, with muscle mass loss in the lower extremity being more pronounced and occurring earlier than in the upper extremity (27). In line with the purpose of our study, only the anterior thigh muscle thickness using USG for its application in the diagnostic algorithm was evaluated. Our findings showed no significant difference between the groups in terms of anterior thigh muscle thickness and STAR measurements.

Our study results for gait speed align with other studies for patients with FMS where gait speed was shown to be reduced significantly (7, 28).

TUG is easy, quick and widely used clinical performance-based measure of lower extremity function, mobility and requires both static and dynamic balance. In studies conducted on patients with FMS, the results related to TUG test vary. In one study involving 20 female patients and a control group of 20 participants with similar age and physical activity levels, no significant difference was found between the two groups in terms of TUG (26). It was suggested that TUG, which is more indicative of balance and agility, might be more closely related to physical activity levels than to FMS symptoms (29). Supporting this finding, this study also found no significant difference in TUG performance between the two groups, which had comparable physical activity levels based on the IPAQ total score.

WPI shows positive correlation with CST in our study, this could mean that more widespread the symptoms, more time taken in CST test. WPI also is negatively correlated with STAR and gait speed, suggesting that with increased symptoms in FMS, muscle thickness is reduced in STAR and gait speed is slower. The negative correlation between SMI and follow-up period could suggest that reduced skeletal muscle mass could be associated with greater duration FMS symptoms.

In this study, a significant positive correlation was found between WPI, SSS, FSS, BDI, FIQ and CST, whereas no similar significance was observed in hand grip strength. This finding, derived from various scales, may suggest that CST, which is also a performance test, provides more meaningful insights than hand grip strength assessment in patients with FMS. It may also support the notion that muscle changes in the lower extremities occur earlier.

The relationship between vitamin levels and sarcopenia parameters in patients with FMS was examined and a significant negative correlation between vitamin D levels and CST was found. This result supports the connection between vitamin D and muscles rich in type II fibers, which are indicative of physical performance (30).

This study has several strengths: the inclusion of a control group, the absence of significant differences between the groups in terms of age and physical activity, as well as vitamin levels that could influence sarcopenia parameters, and the fact that the ultrasound evaluations were conducted by the same experienced radiologist blinded to the group assignments of the participants.

That said, there are certain limitations to the study. Being a cross-sectional study, it does not allow for establishing causal relationships. In addition, the single-center nature of the study, the relatively small sample size, and the inclusion of relatively young patients in the study are possible limitations to the obtaining of more robust results. In line with the objectives of the study, the focus was solely on the measuring of muscle thickness with ultrasound, but other parameters such as muscle cross-sectional area, fascicle length, pennation angle, and echogenicity could have been evaluated to better differentiate between the two groups. Furthermore, our study included only female participants, and future multicenter studies which involve all genders are needed to compare and validate these results.

In conclusion, there is loss of muscle function in female patients with FMS, but loss of muscle mass has not been demonstrated. According to the EWGSOP2 and ISarcoPRM algorithms, the frequency of probable sarcopenia was higher in patient group compared to healthy control group. No statistically significant difference was found between the patient group and the control group in terms of anterior thigh muscle thickness, STAR, and skeletal muscle mass index. It is possible to discuss the risk of probable sarcopenia and dynapenia in the foreground in this population. Further studies are needed to confirm these findings.

## Data Availability

The raw data supporting the conclusions of this article will be made available by the authors, without undue reservation.
